# Molecular analysis of *CYP1B1* in Omani patients with primary congenital glaucoma: a pilot study

**Published:** 2009-07-08

**Authors:** Stefan El-Gayar, Anuradha Ganesh, Gabriela Chavarria-Soley, Sana Al-Zuhaibi, Rayhanah Al-Mjeni, Sandy Raeburn, Alexander A. Bialasiewicz

**Affiliations:** 1 Department of Ophthalmology, Sultan Qaboos University Hospital, Muscat, Oman; 2 Department of Genetics, Sultan Qaboos University Hospital, Muscat, Oman; 3 Institute of Human Genetics, Friedrich Alexander University, Erlangen, Germany

## Abstract

**Purpose:**

To screen cytochrome P4501B1 (*CYP1B1*) for causative mutations in Omani patients with a clinical diagnosis of primary congenital glaucoma (PCG)

**Methods:**

Nine PCG families were recruited for the study. All patients underwent  detailed clinical examinations to confirm the diagnosis of PCG. The families of index patients were also examined. Genealogical information was obtained by pedigree analysis. The primary candidate gene, *CYP1B1*, was amplified from genomic DNA, sequenced, and analyzed in patients to identify the disease-causing mutations.

**Results:**

Eight of the nine PCG families were consanguineous (89%). Molecular analysis of *CYP1B1* showed three distinct mutations, p.G61E, p.D374N, and p.R368H, in seven of nine unrelated PCG index patients (78%). Six patients had homozygous mutations and one had a compound heterozygous mutation. Causative mutations were not identified in two families. In family 4, the index patient was found to be heterozygous for the p.E229K variant. In family 6, although affected individuals were found to be homozygous in the *CYP1B1* region, no mutation could be identified.

**Conclusions:**

This study indicates that *CYP1B1* could be the predominant cause of PCG in the Omani population (78%). Omani PCG patients show allelic heterogeneity. Further studies are needed to delineate the spectrum of *CYP1B1*mutations in Omani PCG families and to identify new or modifier genes contributing to the manifestations of PCG in this region.

## Introduction

Primary congenital glaucoma (PCG; OMIM 231300; gene symbol, *GLC3)* is characterized by congenital elevation of intraocular pressure (IOP) consequent to impaired aqueous outflow via the trabecular meshwork. While uncommon in the West (less than 1 in 30,000 live births), PCG is relatively common in the Middle East with an estimated incidence of 1 in 2,500 live births. This is partly attributed to the high incidence of consanguineous marriages in this region [1]. In Saudi Arabia, a neighboring state of Oman, PCG has been found to be the predominant cause for childhood blindness [1].

Clinically, PCG is unassociated with other ocular or systemic diseases and is divided into three subsets, newborn PCG (patients recognized at birth or in the first month of life), infantile PCG (patients presenting in the first two years of life), and juvenile PCG (patients diagnosed after two years of age) [2]. More than 80% of the cases present within the first year of life with 25% diagnosed in the neonatal period and 60% within the first six months of life [3]. In 75% of cases, both eyes are involved, and males are affected somewhat more often than females [4].

The disease is characterized by high IOP, buphthalmos, megalocornea, and breaks in Descemet’s membrane with corneal opacification. PCG in the Middle East is more aggressive and is associated with poorer therapeutic outcomes than in the West [5,6].

About 10% of all PCG cases are inherited, the mode of inheritance being largely autosomal recessive with variable penetrance. Strong inheritance (vertical transmission) is rarely observed in some families and is explained by pseudodominance [7]. Three chromosomal regions, 2p21 at locus *GLC3A* [8,9], 1p36 at locus *GLC3B* [10], and 14q24.3 at locus *GLC3C* [11] have been reported to be associated with PCG. The only identified gene so far is *CYP1B1* at locus *GLC3A*, and this gene encodes for cytochrome P450 1B1 [8,9]. This enzyme is a cell membrane bound monomeric mixed-function oxygenase believed to interact with an arachidonate metabolite that is important for the normal development and function of the anterior segment of the eye [12].

To date, more than 60 mutations associated with PCG and a few polymorphisms in *CYP1B1* have been found [13], one third of them being deletions or insertions, implying an inherited instability of the gene. A total of six common single nucleotide polymorphisms (SNPs) have been identified in the *CYP1B1* region, one upstream of exon 2 (rs2617266) and five coding SNPs (rs10012 [p. R48G], rs1056827 [p.A119S], rs1056836 [p.V432L], rs1056837 [p.D449D], and rs1800440 [p.N453S]) [11]. These SNPs are embedded in a *CYP1B1* region in linkage disequilibrium [14].

There is a high incidence of consanguinity in Oman (up to 36%), which leads to a high prevalence of autosomal recessive diseases in the country [15]. To date, the genetic basis and prevalence of various mutations among Omani PCG patients has not been studied. We report the results of a pilot study to determine the distribution of *CYP1B1* mutations in Omani patients with PCG.

## Methods

This research was performed in accordance with the Declaration of Helsinki and with the approval of the Medical Research Ethics Committee of the Sultan Qaboos University (Muscat, Oman). The families of nine patients consented to participate after being informed of the nature of the research.

PCG patients who were registered in the ocular genetics database of the Department of Ophthalmology, SQUH, Oman were recalled. Patients with ocular abnormalities or systemic diseases suggestive of secondary glaucoma such as aniridia, anterior segment dysgenesis, Lowe syndrome, and Sturge-Weber syndrome were excluded from the study.

All patients were examined by at least one of the authors (A.G., S.E., S.Z., A.B.), either in the office awake or sedated, or underwent an examination under anesthesia. Ophthalmic examination included evaluation of best-corrected visual acuity (with age-appropriate tests), corneal diameter, IOP (with Tonopen XL or Perkins tonometer), corneal thickness (with ultrasound pachymeter), cup-disc ratio (with indirect ophthalmsocopy with +20D), and axial length (with ultrasound biometry). IOP values were corrected for corneal thickness based on nomograms. Slit lamp examination, gonioscopy, visual field evaluation (automated visual field Humphrey 24–2 or Goldman) and fundus photography were done when feasible. Glaucoma was diagnosed if patients demonstrated an IOP ≥22 mmHg with other signs of PCG including buphthalmos, megalocornea, corneal edema, Haab’s striae, and increased cup/disc ratio.

Genealogical information was obtained by pedigree analysis. The siblings, parents, and other family members suspected to have PCG were clinically examined along with the proband.

Genomic DNA was extracted from peripheral blood. The two coding exons of *CYP1B1* were amplified using four previously published primer pairs [16]. For amplification, a touchdown polymerase chain reaction (PCR) program was used with annealing temperature decreasing from 65 °C to 55 °C over nine cycles followed by 24 cycles with an annealing temperature of 55 °C in a 25 μl mixture (PCR conditions available on request). Sequencing reactions were performed on both strands using the BigDye Terminator Cycle Sequencing Kit v3.1 (Applied Biosystems, Foster City, CA) according to the manufacturer’s instructions. The products were analyzed on an ABI Genetic Analyzer 3730 (Applied Biosystems). Using segregation analysis in the families, haplotypes were constructed using the six previously mentioned common SNPs in the gene, one upstream of exon 2 (rs2617266) and five coding SNPs (rs10012 [p. R48G], rs1056827 [p.A119S], rs1056836 [p.V432L], rs1056837 [p.D449D], and rs1800440 [p.N453S]) [11]. Mutations when found were confirmed in family members.

## Results

Nine patients with PCG (5 males, 4 females) and their families were enrolled in the study. All affected patients had IOPs greater than or equal to 21 mmHg as well as increased corneal diameters (>12mm), cup-disc ratios, and axial lengths. All patients included in the present study had an aggressive form of glaucoma with onset within the first month of life and had undergone multiple surgical interventions for control of their glaucoma. The clinical data are summarized in Table 1 and Table 2.

**Table 1:** Clinical details in study patients with primary congenital glaucoma I.

**Proband**	**Age of onset**	**Age at Dx**	**Current age**	**Corneal Diameter V,H [mm]**	**Corneal Haze OD/OS**	**IOP [mmHg] OD/OS at Dx**	**IOP [mmHg] OD/OS Current**	**cIOP [mmHg] OD/OS**	**Gonioscopy OD/OS [Shaffer] or AC depth**	**Axial Length [mm] OD/OS**	**Refraction [SphericEquivalent] OD/OS**	**Cup-Disc Ratio OD/OS**	**Visual Acuity [Decimal] OD/OS**	**Treatment**
**Family 1**														
proband	at birth	at birth	10 y	12,12/12,12	mild/ mild	ND	19/23	18/22	1–2/1–2	22.74/ 25.17	−1.5/-9.75	0.4/0.8	1.0/0.2	OU Trab (7d); LE ALT+Trab (3y); OU Medications
**Family 2**														
proband	at birth	“late”	24 y	14,14/13,13	clear/ clear	ND	45/45	46/43	3.5 h open, PAS/open	25.23/ 24.04	−3.75/.4.25	1.0/0.9	HM/0.1	OS Medications OS
Affected sibling	at birth	7 y	13 y	11,12/12,15	clear/ severe	ND	18/ digitally soft	18	2/deep AC	ND/ ND	−1.37/NA	0.7/NA	0.1/NLP	OD Trab+MMC (7y); OS no Rx.; OD Medications
Affected sibling	at birth	5 y	11 y	13,15/ND	mild/NA	ND	21/NA	28	1/NA	22.53/ NA	+0.5/NA	0.8/ND	0.5/NA	OD Trab (5y); OS enucleation (9y); OD Medications
**Family 3**														
proband	at birth	5 d	11 y	12.5,12.5 / 12,12	mild / clear	ND	21 /13	10/08	normal AC/normal AC	23.05/ 20.92	0.0/+0.5	0.3/0.4	0.05/0.5	OD Trab (2w) (´3); OS Trab (2w) (´2); OU Medications
**Family 4**														
proband	unclear	unclear	23 y	12.5,12.5/11.5,12.0	clear/ clear	>50/>50	17/15	19/16	3/3	ND/ ND	−3.37/-2.87	0.8/0.9	0.3/0.6	OU Trab (6y); OU Medications
Affected sibling	at birth	at birth	3 y	12.5,13/12.5,12.5; at 14 m: 11,11.5/11,11.5	clear/ clear	>50/>50	12/18	7/14	ND/ND	21.09/ 20.34, at 14 m: 19.92/ 19.67	−1.75/-2.0	0.0/0.0	0.5/0.5	OU Trab+Trab (3y); No medications
**Family 5**														
proband	at birth	at birth	3 y	11,12/ND; at birth: 10,10/12,12	clear / NA	38 / 36	22 / NA	18	normal AC/NA	19.7/ NA, at birth: 18.47/ 19.27	−5.75/NA	0.1/NA	sc 0.05/NLP	OD Trab + Trab (3w); OS Trab + lensectomy+anterior vitrectomy (3w); No medications
**Family 6**														
proband	at birth	at birth	3 y	11.5/12		>30/30	30/25	39/18				0.4/0.5	0.05/0.15 (low coop.)	OU Trab; No medications
Affected sibling	at birth	at birth	6 m	11.5,11.0/10,10	clear / clear	ND			normal AC/normal AC	18.64/ 18.63	−0.62/-1.37	0.5/0.9	follows light/follows light	Medications only; Awaiting Sx
**Family 7**														
proband	at birth	at birth	20 y	8,9/9,11	severe/severe, at birth: BE “white cornea”	ND	NA/NA		NA/NA	NA/NA	NA/NA	NA/NA	NLP/NLP	OU Trab (9d) (x1) (5y); No medications
Affected sibling	at birth	at birth	18 y	-,17/-,17	severe/severe, at birth: BE “white cornea”	ND	BE digitally hard		NA/NA	>30/ >30	NA/NA	NA/NA	LP/LP	OU Trab (3–4y); No medications
Affected sibling	at birth	at birth	8 y	-,14.5/-,14.5	severe/severe, at birth: BE “white cornea”	ND	BE digitally hard		deep AC/deep AC	≈27/ ≈25	NA/NA	NA/NA	LP/LP	OU Trab (3–4m); No medications
**Family 8**														
proband	unclear	12 y	26 y	12.5,12.5/12,12	mild/clear	ND	45/16	39/18	deep AC/deep AC	27.88/ 25.57	−11.9/-1.68	NA/1.0	LP/HM	OS Trab (22y); No medications
Affected sibling	< 1 y	1 y	23 y	14,14/13.5,14.5	clear / mild	ND	26/21	17/15	normal AC/normal AC	29.10/ 22.36	≈-6.5/NA	0.9/NA	0.075/NLP	OS Trab (1year); No medications
Affected sibling	at birth	at birth	18 y	12.5,12.5/13,13	severe/severe	ND	digitally soft/ digitally hard		NA/NA	≈13/ ≈25	NA/NA	NA/NA	NLP/NLP	OU Trab (20d);
**Family 9**														
proband	1 m	1 m	17 y	10,10/19,19	severe/severe	ND	BE digitally soft		NA/NA	≈21/ ≈29	NA/NA	NA/NA	NLP/NLP	OS Trab (x7); No medications

All data refer to time of last examination except where stated differently. ALT – argon laser trabeculoplasty, d: day(s), Curr.: current, Dx: diagnosis, cIOP: pachymetry corrected intraocular pressure, H: horizontal, HM: hand movements, IOP: intraocular pressure, LP: light perception, m: month(s), MMC: mitomicin C, NLP: no light perception, w: week(s), NA: not applicable, ND: no data available, OD: right eye, OS: left eye, OU: both eyes, PAS: peripheral anterior synechia, Rx: Treatment, Sx: surgery, Trab – Trabeculectomy, Trab+trab: Trabeculectomy + Trabeculotomy, V-vertical, y: year(s).

**Table 2:** Clinical details in study patients with primary congenital glaucoma II.

**Clinical Parameter**	**Data Range (n=18 PCG patients)**
Age of onset	at birth–1 month*
Age at diagnosis	at birth–12 years
Age currently	6 months–26 years
Corneal diameter horizontally [mm]	9–19
Intraocular pressure [mmHg]	12–45
Cup-disk ratio	0.0–1.0
Axial length [mm]	18.47->30 (and phthisis bulbi in some cases)
Pachymetry central [µm]	465–816
Refraction as spherical equivalent [D]	+0.5 to −11.9
Visual acuity [decimal]	No Light Perception–1.0

All data refer to time of last examination except where stated differently. The asterisk denotes that in two PCG patients, the age of onset was unclear (but definitely within one year of age). PCG-primary congenital glaucoma.

**Figure 1:**
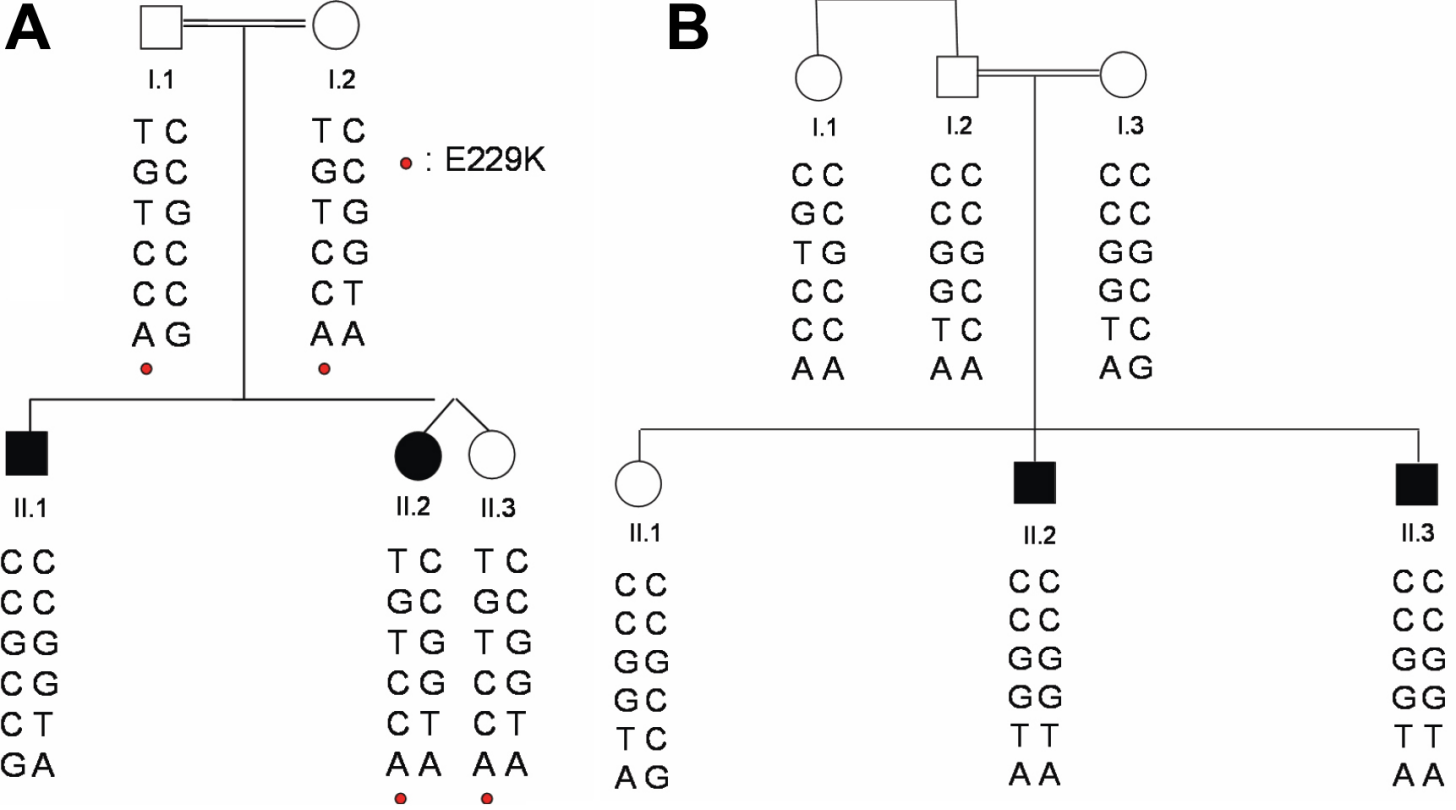
Pedigree with haplotypes of family 4 (heterozygous mutation). **A**: Pedigree with haplotypes of family 4 (heterozygous mutation) is shown. In this family, the index patient (II.2) is heterozygous for the E229K mutation as is her unaffected dizygotic twin (II.3) and parents (I.1 and I.2). On the other hand, the index patient’s brother (II.1) does not carry the E229K mutation but has the disease. This suggests that the E229K mutation cannot be causative for PCG in this family. The order of the SNPs from top to bottom is rs2617266, rs10012 (p. R48G), rs1056827 (p.A119S), rs1056836 (p.V432L), rs1056837 (p.D449D), and rs1800440 (p.N453S). **B**: Pedigree with haplotypes of Family 6 (no mutation) is shown. In this family, no *CYP1B1* mutation was identified. Analysis of the six known SNPs in *CYP1B1* revealed homozygosity for the 5′-CCGGTA-3′ haplotype in both PCG-affected brothers (II.2 and II.3) and heterozygosity in the unaffected sister (II.1) and parents (I.2 and I.3). This indicates a critical role for the 5′-CCGGTA-3′ haplotype in PCG. The order of the SNPs from top to bottom is: rs2617266, rs10012 (p. R48G), rs1056827 (p.A119S), rs1056836 (p.V432L), rs1056837 (p.D449D), and rs1800440 (p.N453S).

Consanguinity was found in eight of the nine families (89%) without any gender preference (Table 3). In five families, more than one individual was affected. The presumed mode of inheritance according to pedigree analysis was autosomal recessive in all families. Molecular analysis of *CYP1B1* permitted the identification of three causative mutations in seven of the nine index cases (78%; Table 3). The p.G61E, p.D374N, and p.R368H mutations have all been previously reported as disease-causing mutations [1,17]. The identified mutations were homozygous in six patients and compound heterozygous in one patient (Table 3). The mutations segregated with the disease in the seven families. The index case in family 4 presented the previously reported risk-associated but not clearly disease-causing [18] p.E229K variant in heterozygous form. Haplotype analysis for family 4 using the six coding SNPs in *CYP1B1* ruled out this variant as the cause of PCG (Figure 1A). It did not segregate with the disease since the affected brother of the index patient (II.1) did not carry the variant. No mutation in *CYP1B1* could be identified for family 6 (Table 3). Haplotype analysis, however, suggested linkage to the *CYP1B1* locus, *GLC3A* (Figure 1B). Both affected individuals (II.2 and II.3) were homozygous for the same SNP haplotype, 5′-CCGGTA-3′, while their unaffected sister was heterozygous. Thus, a total of 14 mutations and one risk-associated variant were found in the nine patients representing an overall mutation rate of 14 in 18 (78%) studied chromosomes.

**Table 3:** *CYP1B1* mutations associated with primary congenital glaucoma in Omani patients.

**Family number**	**Consanguinity**	**Number of affected individuals**	**Gender**	**Mutations**		**Exon**
				**Allele 1**	**Allele 2**	
1	No	1	Male	p.R368H	p.R368H	III
2	Yes	4	Female	p.D374N	p.D374N	III
3	Yes	1	Female	p.G61E	p.G61E	II
4	Yes	2	Female	p.E229K	no mutation	II
5	Yes	1	Male	p.G61E	p.R368H	II/III
6	Yes	2	Male	no mutation	no mutation	
7	Yes	3	Male	p.D374N	p.D374N	III
8	Yes	4	Female	p.G61E	p.G61E	II
9	Yes	1	Male	p.R368H	p.R368H	III

Consanguinity was found in eight of the nine families (89%). In five families, more than one individual was affected. Molecular analysis of *CYP1B1* permitted the identification of three causative mutations in seven of the nine index cases (78%).

## Discussion

PCG is a genetically heterogeneous disorder that maps to at least three different loci. However, in the majority of PCG patients, the candidate locus has been found to be *GLC3A*, which codes for a cytochrome p450 protein called CYP1B1 [9]. Among patients with PCG, the proportion of patients due to *CYP1B1* mutations is variable across populations from ~100% among Saudi Arabians [1,17] and Slovakgypsies (Roms) [19] to ~20% in Japanese [20]. We detected three distinct disease-causing *CYP1B1* mutations in seven of the nine PCG index patients (78%). Our results indicate that *CYP1B1* mutations play a crucial role in Omani PCG patients as in other Arab populations.

The worldwide profile of variations in the coding region of *CYP1B1* in patients with PCG thus far reported is heterogeneous and includes ~70 alterations (Human Genome Mutation Database) [21]. However, the Slovakgypsies (Roms) and Saudi Arabian patients with PCG exhibit allelic homogeneity that has largely been attributed to consanguinity and inbreeding [17,19]. The detection of three disease-causing mutations in seven index cases indicates that Omani PCG patients show allelic heterogeneity. The three mutations we found in Omani patients (p.R368H, p.D374N, and p.G61E) have been reported as the most common mutations in the Saudi population, accounting for 72%, 12%, and 7% of the tested alleles, respectively [1,17]. Oman has been a major gateway in human history with evidence of clear contacts with sub-Saharan West Africa, East Africa (Zanzibar/Mombasa), Iraq, Iran, Pakistan, and India and is expected to have a rich genetic legacy. The degree of heterogeneity within different populations as well as the distribution of mutations have been seen to be very variable and are believed to be attributable to variations in gene pools among the different populations [22].

Among the seven index cases with *CYP1B1* mutations, one carried two different mutations (compound heterozygous). The large proportion of Omani PCG patients (6/7; 86%) carrying homozygous mutations in *CYP1B1* is indicative of extensive consanguineous marriages in the Omani population.

The p.E229K variant found in family 4 did not segregate with the disease (Table 3, Figure 1A). Haplotype analysis suggested that mutations in a locus different than *GLC3A* could be responsible for PCG in this family. In family 6, (Table 3, Figure 1B) no mutation could be identified. However, both affected individuals were homozygous in the *CYP1B1* region, making it likely that a mutation in the promoter or in some other regulatory region of the gene is responsible for the disease. Further work with family 4 (performing a linkage analysis after recruiting additional affected and healthy family members) and family 6 (analysis of cDNA in the affected family members) is in progress.

Our study is the first report of molecular genetic analysis of PCG in the Omani population. To verify the results of this pilot study, to further delineate the role of *CYP1B1* mutations in the pathogenesis of PCG in the Omani population, and to identify new or modifier genes contributing to the manifestations of PCG in this region, further studies with a larger sample of patients are being planned. Identifying the mutation spectrum of *CYP1B1* that causes PCG in the Omani population has implications in devising molecular diagnostics for rapid screening in predisposed families that would aid in early intervention.
